# Synthesis and characterization of epoxy resin of 9,9′-bis-(3,5-dibromo-4-hydroxyphenyl) anthrone-10 and its jute composite

**DOI:** 10.1080/15685551.2017.1421008

**Published:** 2018-01-30

**Authors:** Jabal D. Thanki, Jignesh P. Patel, P. H. Parsania

**Affiliations:** ^a^ Polymer Chemistry Division, Department of Chemistry, Saurashtra University, Rajkot, Gujarat, 360 005, India

**Keywords:** Epoxy resin, thermal stability, tensile strength, flexural strength, electric strength, volume resistivity, hydrolytic stability

## Abstract

Epoxy resin of 9,9′-bis-(3,5-dibromo-4-hydroxyphenyl) anthrone-10 (EANBr, EEW 490) was synthesized and was characterized by IR and ^1^HNMR . EANBr and EPK3251 cured resin (EANBrC) were characterized by DSC and TGA at 10°Cmin^−1^ under nitrogen atmosphere. Broad DSC endothermic transitions of EANBr (265.3 °C) and EANBrC (291.4 °C) are due to some physical change and further confirmed by no weight loss in their TG thermograms. EANBr and EANBrC are thermally stable up to 340 °C and 310 °C, respectively. EANBr has followed single step degradation kinetics, while EANBrC has followed two step degradation kinetics. EANBr followed apparently zero order kinetics, while EANBrC followed apparently second order (1.80) and first order (0.89) degradation kinetics, respectively. Ea and A values of EANBrC (299.7 kJmol^−1^ and 6.32 × 10^20^ s^−1^) were found higher than that of EANBr (201 kJmol^−1^ and 2.45 × 1013 s^−1^) due to more rigid nature of EANBrC. The ΔS^*^ value of the first step degradation of EANBrC (146.3 JK^−1^ mol^−1^) was found much more than that of EANBr (4.6 JK^−1^ mol^−1^). Jute – EANBr composite (J-EANBr) was prepared by compression molding technique at 120 °C for 5 h and under 20 Bar pressure. The observed tensile strength, flexural strength, electric strength and volume resistivity of J-EANBr are 24.7 MPa, 19.0 MPa, 1.8 kVmm^−1^ and 3.5 × 10^12^ ohm cm, respectively. Water absorption in J-EANBr was carried out at 30 ± 2 °C against distilled water, 10% NaCl, 10% HCl, 10% HNO_3_, 10% H_2_SO_4_, 10% NaOH, and 10% KOH and also in boiling water. The equilibrium time and equilibrium water content for J-EANBr in different environments are 384–432 h; 12.7–15.2%, respectively. The observed equilibrium water content and diffusivity trends of J-EANBr are KOH>H_2_SO_4_>HCl>NaOH>H_2_O>NaCl and H_2_O>NaCl>NaOH>H_2_SO_4_>HCl>KOH, respectively. Good thermo-mechanical, electrical properties and excellent hydrolytic stability of J-EANBr may be useful for high temperature applications in diverse fields.

## 1. Introduction

The increasing use of polymeric materials in building, transports, electronic and computer equipment demands brominated epoxy resins as flame retardants. Bromine and chlorine containing epoxy resins release corrosive and obscuring smoke, halogenated dibenzodioxines and dibenzofurans super toxic compounds on burning. Such compounds are hazardous to environment and human health [1–4]. For electronic information industry, high thermal conductivity, high thermal stability, low coefficients of thermal expansion and low dielectric constant of substrate and packaging materials are essential. The brominated epoxy resins are most widely used as flame retardant in printed circuit boards [5,6]. The polymeric nature and chemical structure of brominated epoxy resin offers several advantages such as high thermal stability and thermal aging, high UV stability, excellent processability and noncorrosive nature.

Yin et al. [6] have investigated the thermal degradation of brominated epoxy resins by hydrothermal technology and the compositions of the degradation products such as phenol, o-cresol, p-cresol and other low molecular mass compounds without bromine as degradation products of brominated epoxy resin were determined by GC-MS.

Luda et al. [7] have investigated the thermal degradation of the brominated epoxy resin in inert atmosphere using Thermogravimetry (TG), high resolution thermogravimetry (HRTG), Infrared (IR), and Gas Chromatography–Mass Spectrometry (GC-MS) of gases and high boiling degradation products (HBP). Three step thermal degradation was found due to brominated and non-brominated parts of the epoxy resin and char formation. Brominated aliphatic compounds, mono- and dibromo phenols were released in the first step, while non-brominated part of brominated epoxy resin released non-substituted and alkyl substituted phenols, bisphenol A, alkoxy aromatics in the second step. The possible ways of the product formation are predominantly based on homolytic processes. The nitrogen-containing group accumulate in the residue due to the high level of crosslinking whereas unsaturated cycles contribute to the charring of the residue.

Luda et al. [8] and Balabanovich et al. [9] have investigated the effect of different curing agents on thermal degradation of brominated epoxy resins. Nitrogen containing different hardeners strongly affected the thermal degradation behavior of the brominated epoxy resins. They analyzed volatile degradation products such as phenol, isopropyl- and isopropenyl phenol, mono- and di-bromophenols, bisphenol A, mono-, di-, tri- and tetra-bromobisphenol-A by Pyrolysis-Gas Chromatography-Mass Spectroscopy (PY-GC-MS) at 423 °C. No nitrogen containing volatile products or HBr were found. They found variation of 30–60 °C in thermal stability of cured epoxy resins depending on the hardener. They suggested a cooperative action of bromine and nitrogen in chain scission of epoxy resins. The ability of the hardener in fixing HBr, evolution from tetrabromo bisphenol-A units was found to depend on the basicity of the N atom of the hardener. They concluded that the lower is the basicity, the lower is the scavenging effectiveness and hence the higher is the thermal stability.

Brominated epoxy resins are thermally less stable than non-brominated ones [7,10]. The degradation of brominated epoxy resins yields very toxic products as confirmed by micro-scale pyrolysis [7], or pyrolysis and laser ablation mass spectrometry [11]. The onset of degradation reaction is associated with the formation of hydrogen bromide, which further destabilizes the epoxy network. It is generally accepted [12,13] that degradation of epoxy resins starts by dehydration of secondary alcoholic groups followed by homolytic scission of the formed allylic bond. Repetition of bond scission of the epoxy network leads to the evolution of the low molecular mass high boiling degradation products, whereas polymerization of unsaturated bonds resulting from dehydration and subsequent aromatization contributes to charring [13].

To the best of our knowledge no work has been reported on synthesis and characterization of epoxy resin of 9,9′-bis-(3,5-dibromo-4-hydroxyphenyl) anthrone-10 (EANBr). In the present investigation we have reported synthesis of EANBr and its characterization by epoxy equivalent, spectral and thermal techniques, Also preparation and evaluation of mechanical and electrical properties and chemical resistance of Jute-EANBr composite against water, acids, alkalis and salt.

## 2. Experimental

### 2.1. Materials

1,4-Dioxane, tetrahydrofuran (THF), methylethylketone (MEK), chloroform, 1,2-dichloromethane were purified according to reported methods [14]. Epichlorohydrin, isopropyl alcohol (IPA), N,N′-dimethylformamide, dimethyl sulfoxide, methanol, pyridine, etc. were used as received. Woven jute fabric (Brown jute, Corchorus capsularis) was purchased from local market, respectively. EPK-3251 was supplied by Berry Plastics Pvt. Ltd. Manjusar, Dist. Vadodara, Gujarat. Mylar film was used as a mold releasing agent.

### 2.2. Synthesis of epoxy resin of 9,9′-bis-(3,5-dibromo-4-hydroxyphenyl) anthrone-10 (Scheme 1)

A 2-liter 3-neck round bottomed flask was equipped with a mechanical stirrer, thermometer, and reflux condenser. To this flask 0.5 mol (347 g) of 9,9′-bis-(3,5-dibromo-4-hydroxyphenyl) anthrone-10, 2.5 mol (196 ml) of epichlorohydrin and 200 ml of IPA were added and the mixture was heated to 70 °C. 1 Mol sodium hydroxide was dissolved in 70 ml water and was added dropwise over a period of 30 min, while maintaining the reaction temperature at 70 °C. After the completion of sodium hydroxide addition, the reaction mass was refluxed for 1 h and cooled to the room temperature. Reddish brown resin was isolated from water, filtered, washed well with distilled water and dried. The crude resin was extracted in chloroform and evaporated to dryness. The resin is soluble in common organic solvents such as 1,4-dioxane, tetrahydrofuran (THF), methylethylketone (MEK), 1,2-dichloromethane, N,N′-dimethyl- formamide, dimethyl sulfoxide, chloroform, etc. Epoxy equivalent weight was determined by pyridiniumchloride method and it was 490.

### 2.3. Preparation of jute -EANBr composite

Into a 500 ml beaker containing 100 ml tetrahydrofuran, 180 g of EANBr and 25.24 g stoichiometric amount of EPK 3251 were dissolved at room temperature. Resultant solution was applied to jute (350 GSM) fabric (24 cm × 24 cm) with a smooth brush and solvent was allowed to evaporate at room temperature. Eight such impregnated sheets were stacked one over the other between two mylar films. The stacked plies were kept between two teflon sheets and were kept between two preheated stainless plates and pressed under 20 bar pressure at 120 °C for 5 h and 2 h at room temperature. Silicone spray was used as a mold releasing agent. Here after jute composite is designated as J-EANBr. Bleeded cured resin was collected and used for its thermal analysis. Samples with required dimensions were machined for mechanical and electrical tests. For chemical resistance test, samples of 3 cm × 3 cm were machined and their edges were sealed with matrix material.

## 3. Measurements

Epoxy equivalent of EANBr was determined by pyridiniumchloride method [15]. The FTIR spectrum of EANBr was scanned on a Shimadzu 1S-IR affinity FTIR spectrometer over the frequency range from 4000 to 600 cm^−1^. The ^1^H NMR and ^13^CNMR spectra of EANBr were scanned on a Bruker AVANCE II (400 MHz) spectrometer by using CDCl_3_ as a solvent and TMS as an internal standard. Differential Scanning Calorimetric (DSC) and Thermo Gravimetric (TG) thermograms of EANBr and cured EANBr (EANBrC) were scanned on a Shimadzu DSC-60 and Shimadzu DTG-60H, respectively at 10 °C min^−1^ heating rate in nitrogen atmosphere (flow rate 100 ml min^−1^) using standard aluminum pans. Known mass of the samples were taken in aluminum pans covered by empty aluminum lids and sealed using a crimper. The DSC and TG thermograms were scanned over the temperature range from 40 to 350 °C and 40 to 700 °C, respectively. Tensile (ASTM-D 638-01) and flexural (ASTM D-790-03) strengths of J-EANBr was measured on a Shimadzu Autograph Universal Tensile Testing Machine, Model No. AG-X Series at a speed of 10 mm min^−1^. Electric strength (IEC-60243-Pt-1-1998) and volume resistivity (ASTM-D-257-2007) measurements were made on a high voltage tester (Automatic Electric-Mumbai) in air at 27 °C by using 25/75 mm brass electrodes and a Hewlett Packard high resistance meter in air at 25 °C after charging for 60 s at 500 V DC applied voltage at ERDA Vadodara,Gujarat. Replicate measurements (3–5) on each sample were performed and average values were considered. Water absorption study was carried out at room temperature (30 ± 2 °C) according to ASTM- D570-98 by a change in mass method against distilled water, 10% NaCl, 10% HCl, 10% HNO_3_, 10% H_2_SO_4_, 10% NaOH, and 10% KOH. Pre weighed samples were immersed in distilled water, 10% NaCl, 10% HCl, 10% HNO_3_, 10% H_2_SO_4_, 10% NaOH, and 10% KOH solutions at room temperature. Samples were periodically taken out from the solutions, wiped surfaces with tissue papers on both the sides, reweighed and reimmersed in the solutions. The process was carried out till equilibrium was established.

## 4. Results and discussion

### 4.1. Spectral analysis

**Figure 1.**
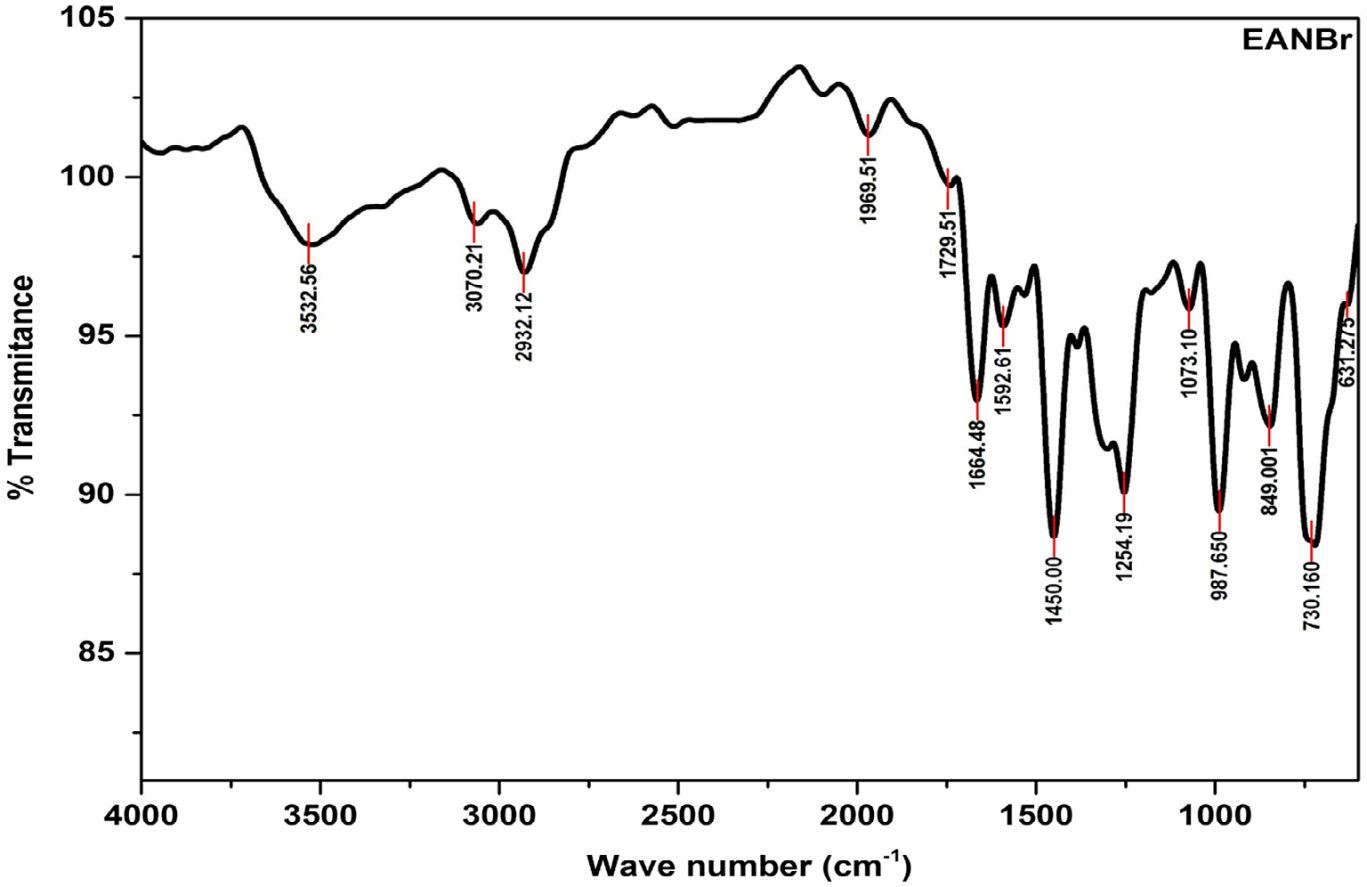
FTIR spectrum of epoxy resin of 9, 9’-bis (3,5-dibromo-4-hydroxyphenyl) anthrone-10 (EANBr).

^1^HNMR spectrum of EANBr is presented in Figure 2A. Different types of protons, their chemical shifts (ppm), and multiplicities are assigned as follows: 1.65 [s, –CH–OH, (h), 1.35], 2.72 [d, –CH–CH_2_, (k), 1.71], 2.89 [d, –CH–CH_2_, (l), 1.63], 3.46 [m, –CH(OH), (g), 1.53], 4.02 [d, –OCH_2_, (j), 1.65], 4.11 [d, –CH_2_O, (I, f) 2.05], 7.05 [m, –ArH, (a, b), 6], 7.51 [m, –ArH, (c, d), 4.38], 8.30 [d, –ArH, (e), 2.09]. Residual CDCl_3_ appeared at about 7.23.

IR spectrum of EANBr is presented in Figure 1. The characteristic IR absorption frequencies (cm^−1^) are assigned as follows: 3532.56 (–OH str.), 3070.21(=C–H str.), 29332.12 (C–H_,_ asym., str.), 1664.48 (–C=O, str.), 1592.61 (Ar C=C, str.), 1450 (Alkane C–H, def.), 1254.19 (Ar–O–R str.), 1071.1 (C–O–H, str.), 987.65 and 849 (C–H, oopd.), 730.14, 631.2 (C–Br, str.).

**Figure 2A.**
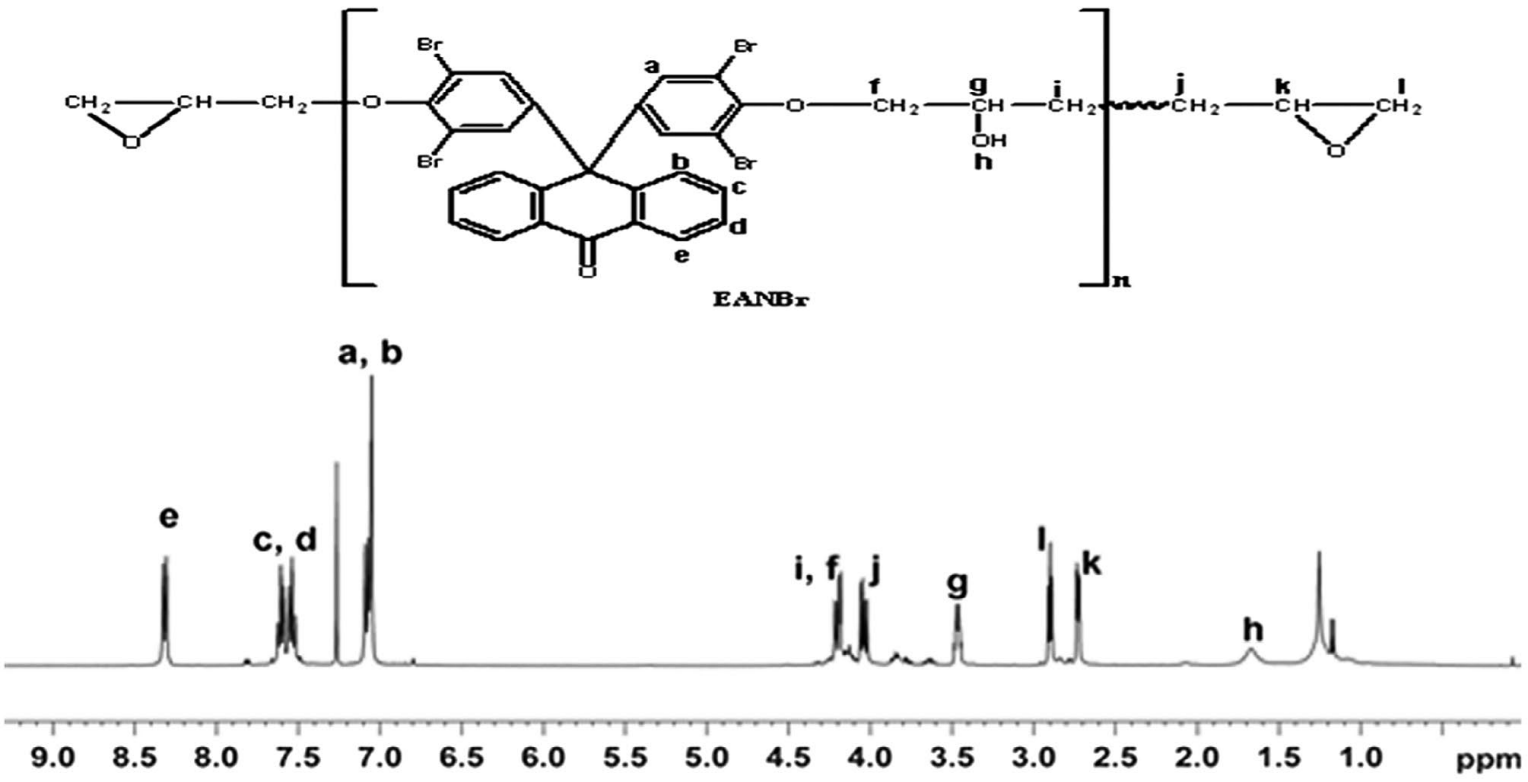
A ^1^HNMR (400 MHz) spectrum of epoxy resin of 9,9′-bis(3,5-dibromo-4-hydroxy phenyl) anthrone-10 (EANBr) in CDCl_3_.

^13^CNMR spectrum of EANBr is presented in Figure 2B. Different types of carbons and their chemical shifts (ppm) are assigned as follows: 45.52 (a), 50.09 (j), 56.46 (b), 74.30 (c, d, e), 118.27 (g), 128.40 (n), 130.05 (l, o), 132.09 (p), 133.42 (m), 133.99 (h), 144.12 (i), 146.60 (k), 152.06 (f) and 183.74 (q). Residual CDCl_3_ appeared at about 77.40–76.77. Thus, IR and NMR spectral data supported the structure of EANBr.

**Figure 2B.**
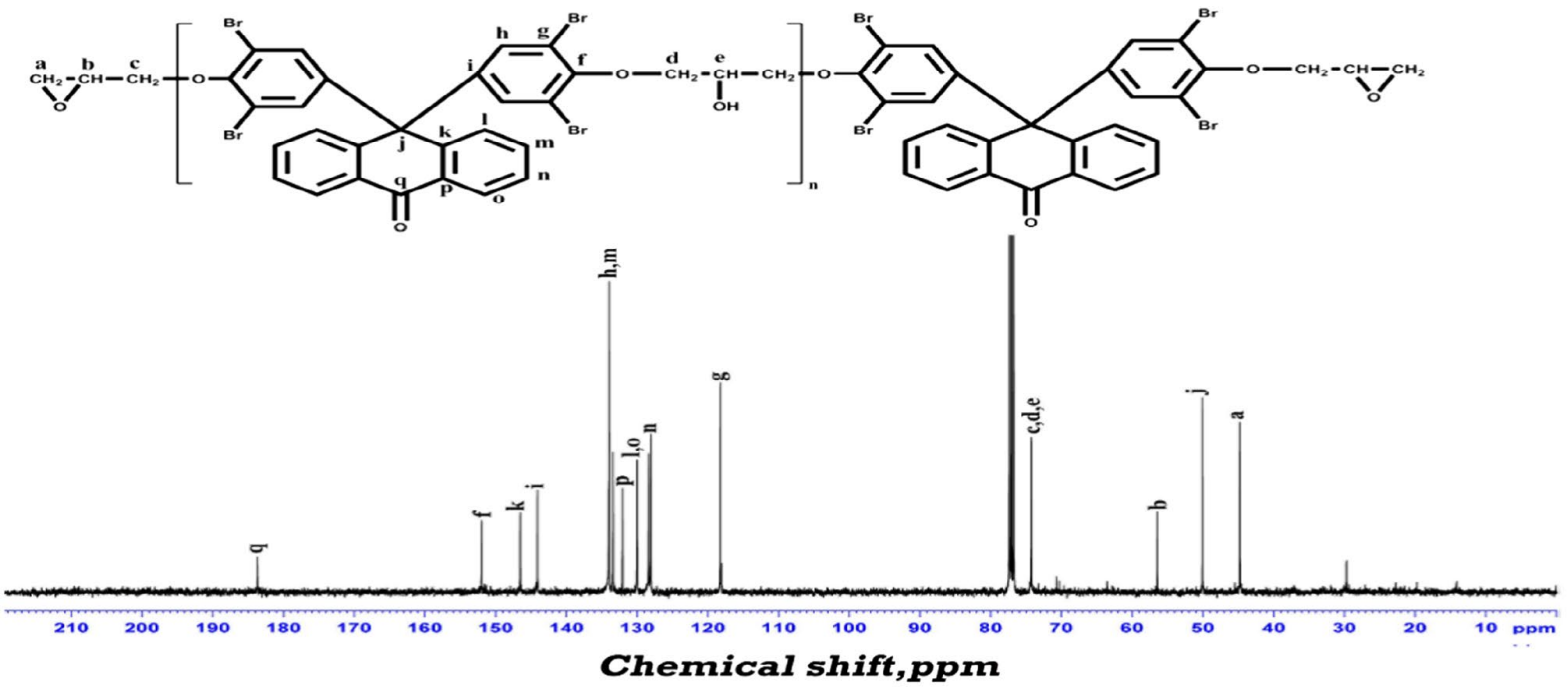
^13^CNMR (400 MHz) spectrum of epoxy resin of 9,9′-bis(3,5-dibromo-4-hydroxy phenyl) anthrone-10 (EANBr) in CDCl_3_.

### 4.2. Thermal analysis

**Figure 3.**
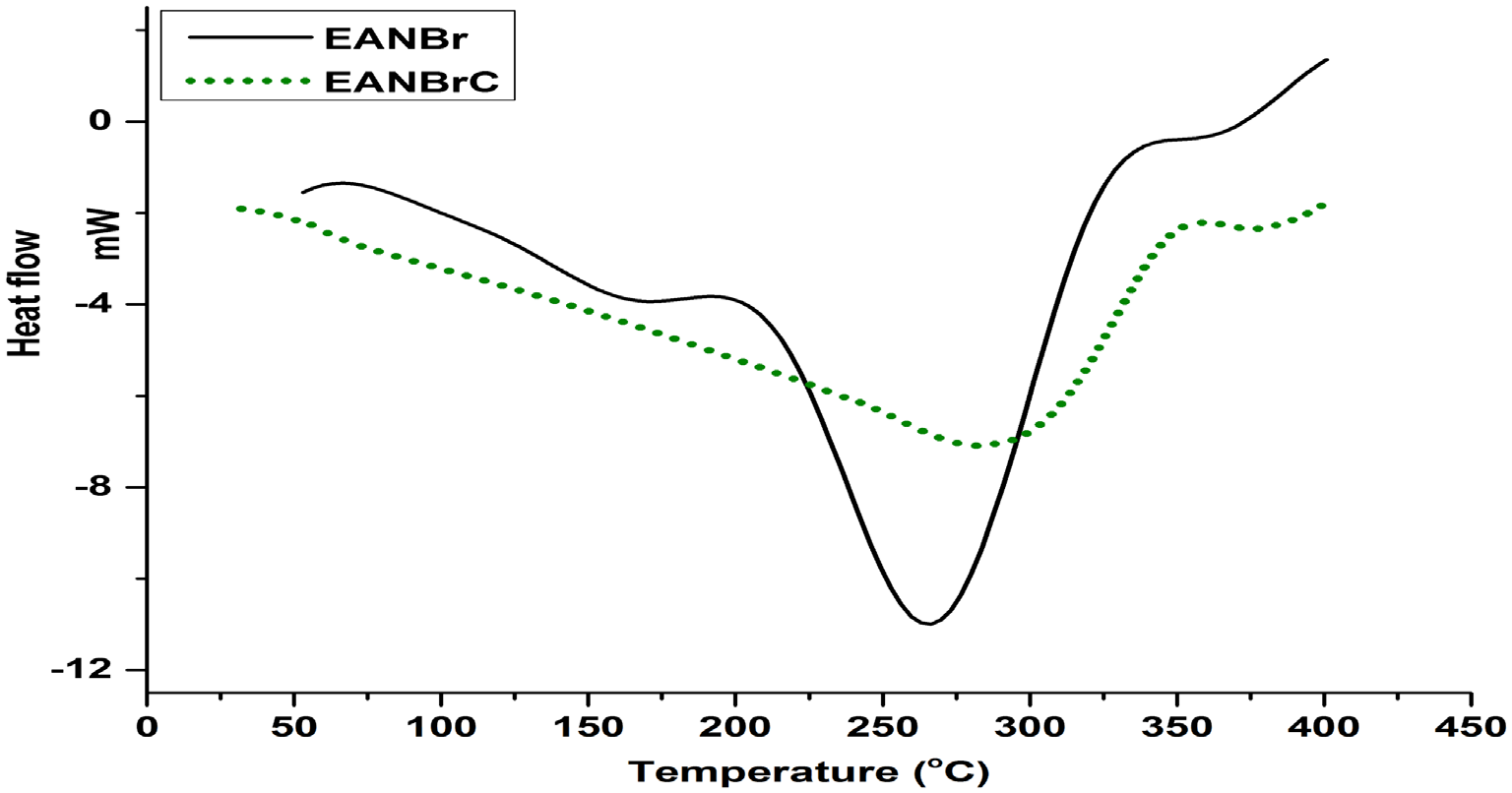
DSC thermograms of EANBr and EANBrC at 10 °C min^−1^ heating rate in nitrogen atmosphere.

**Figure 4.**
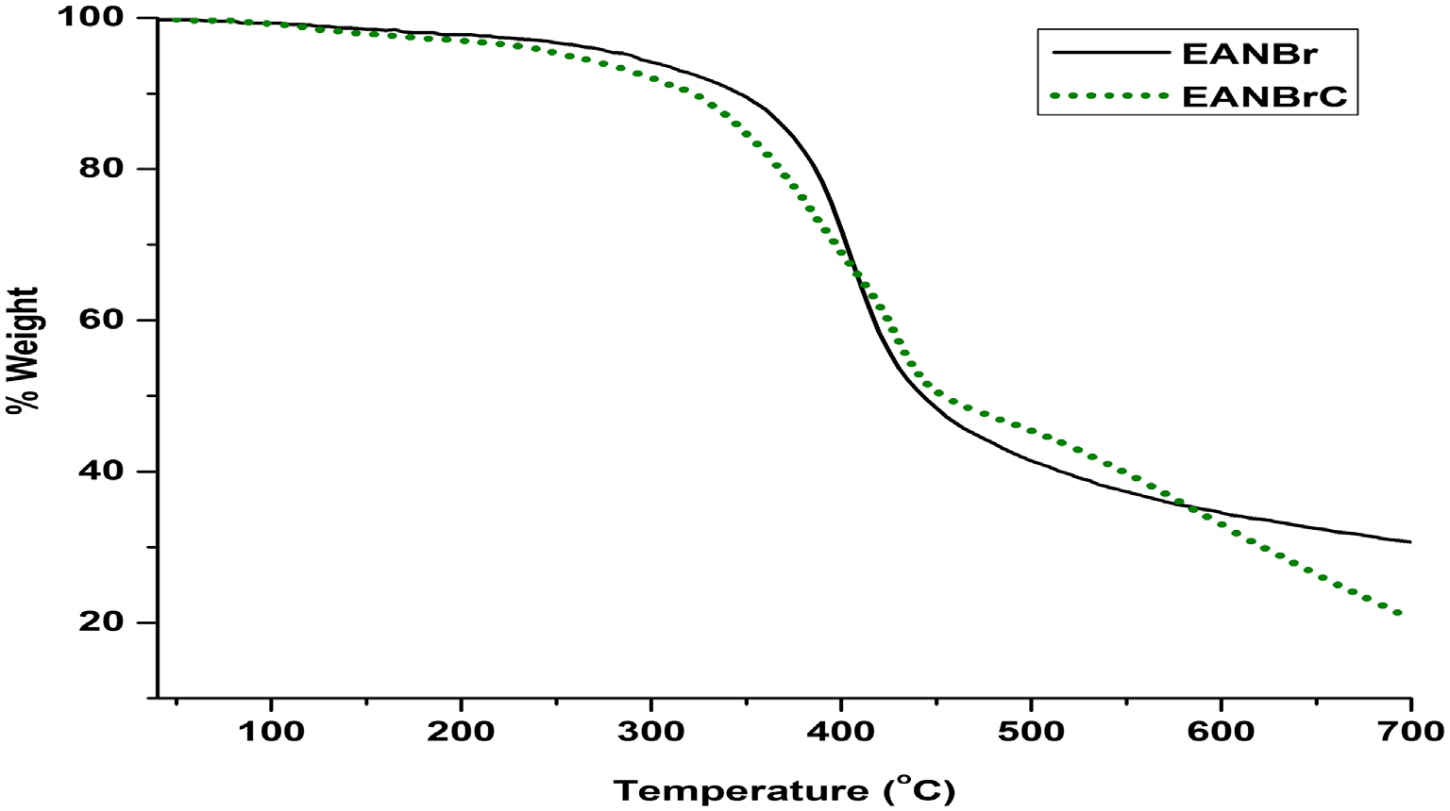
TG thermograms of EANBr and EANBrC at 10 °C min^−1^ heating rate in nitrogen atmosphere.

DSC thermograms of EANBr and EANBrC are presented in Figure 3. The observed broad endothermic transition of EANBr (265.3 °C) and EANBrC (291.4 °C) are due to some physical change and further confirmed by no weight loss in corresponding TG thermogram (Figure 4). From Figure 4, it is clear that EANBr has followed single step degradation reaction, while EANBrC has followed two step degradation reactions. EANBr and EANBrC are thermally stable up to 340 °C and 310 °C, respectively. As compared to EPK 3251cured EAN (360 °C) [16], EPK 3251 cured EANBr (310 °C) has shown lower thermal stability in accordance to literature report [7,10]. Initial decomposition temperature (*T*_o_), decomposition range, temperature of maximum weight loss (*T*_max_), % weight loss and % residue remained at 700 °C for EANBr and EANBrC are presented in Table 1. The *T*_max_ values were derived from dW/dt against Temperature plots. The *T*_max_ value for EANBrC (416.5 °C) is considerably more than that of EPK 3251 cured EAN (394.4 °C)[16]. EANBrC (416.5 °C) has shown somewhat higher value of *T*_max_ than that of EANBr (407.1 °C).

**Table 1.** TGA data of EANBr and EANBrC.

Sample	*T*_o_, (^o^C)	Decompn. range, °C	*T*_max_, °C	% Wt. loss	% Residue at 700 °C
EANBr	340	340–468	407.1	45.1	30.7
EANBrC	310	310–475	416.5	17.6	20.4
		490–660	585.2	38.5	

The associated kinetic parameters such as energy of activation (E_a_), frequency factor (A) and order of the reaction (n) for both the resins were derived according to Anderson-Freeman method [17]:

(1) $$\Delta \frac{\text{lnd}W}{\text{d}t}=n\Delta \text{ln}W\;-\;\frac{E_{a}}{R}\Delta \left(\frac{1}{T}\right)$$

(2) $$A=\;\frac{E_{a}\beta }{RT^{2}}e^{\frac{E_{a\beta }}{\mathrm{RT}}}$$

(3) $$\Delta S^{*}=R\text{ln}\frac{\mathrm{Ah}}{\mathrm{kT}}$$

where d*W*/d*t* is the weight loss with time, *W* is the active weight of the substance, *β* is the heating rate*, R* (8.314JK^−1^ mole^−1^) is the gas constant, *h* (6.626 × 10^−34^Js^−1^) is the Planck’s constant, *T* is the temperature and *k* (1.380 × 10^−23^JK^−1^ mol^−1^) is the Boltzmann constant. The least square values of above mentioned parameters along with regression coefficients (*R* ^2^) are reported in Table 2. From Table 2, it is observed that apparently EANBr has followed zero order (0.12) degradation kinetics, while the first and second steps of EANBrC followed apparently second order (1.80) and first order (0.89) degradation kinetics, respectively. Considerably EANBrC (299.7 kJmol^−1^ and 6.32x10^20^ s^−1^) has shown higher Ea and A values than that of EANBr (201kJmol^−1^ and 2.45 × 1013 s^−1^) indicated more rigid nature of EANBrC. The Ea value of EANBrC for first step (299.7 kJmol^−1^) is almost doubled than that of first step (147.2 kJmol^−1^). Similarly the value of ΔS^*^ of the first step (146.3 JK^−1^ mol^−1^) of EANBrC is much more than that of EANBr (4.6 JK^−1^ mol^−1^). The ΔS^*^ value for second step (−128.1JK^−1^mol^−1^) of EANBrC is negative and large in magnitude. The large and positive magnitude of ΔS^*^ indicated that the transition state is in disorderly state and vice versa. EANBr (30.7%) has shown considerably higher amount of residue at 700 °C than that of EANBrC (20.4%), which is much smaller than EPK 3251 cured EAN (52.5%) [16]. Degradation of polymers is a complex process and involves a variety of reactions. Degradation starts from weak points with the formation of free radicals, which further undergo reactions. Considerable amount of residue remained at 700 °C indicated the formation of highly thermally stable product, which may furtherdegrade at elevated temperature.

**Table 2.** Kinetic parameters of EANBr and EANBrC.

Sample	E_a_, kJmol^−1^	A, s^−1^	ΔS* , JK^−1^ mol^−1^	*n*	*R*^2^
EANBr	201	2.45 × 10^13^	4.6	0.12	09961
EANBrC	299.7	6.32 × 10^20^	146.3	1.80	0.9964
	147.2	3.64 × 10^6^	−128.1	0.89	0.9941

Note: ΔS* is the change in entropy of activation.

### 4.3. Mechanical and electrical properties

A comparative mechanical and electrical properties of J-EANBr and J-EAN [16] are reported in Table 3 from which it is observed that J-EANBr has shown good studied properties. J-EANBr has shown almost comparable tensile strength with that of J-EAN (23.8 MPa) and somewhat lower flexural strength (21.6 MPa) [16]. J-EANBr has shown 40% lower electric strength than J-EAN (3 kVmm^−1^) but it has shown 29 times better volume resistivity than J-EAN (1.2x10^11^ohm cm) [16]. Observed differences in mechanical and electrical properties of J-EANBr and J-EAN are mainly due to different degrees of cross linking and different polarity of the two matrices, which influences interfacial adhesion. Better volume resistivity of J-EANBr is due to cancellation of partial charges of EANBr with OH groups of jute fibers in forming better interfacial adhesion. Comparatively good mechanical and electrical properties of J-EANBr signifies its importance for low load bearing housing, electrical and electronic applications.

**Table 3.** A comparative mechanical and electrical properties of J-EANBr and J-EAN.

Composite	Tensile strength, MPa	Flexural strength, MPa	Electric strength, kVmm^−1^	Volume resistivity.ohm cm
J-EANBr	24.7	19.0	1.8	3.5 × 10^12^
J-EAN [16]	23.8	21.6	3.0	1.2 × 10^11^

### 4.4. Water absorption

**Figure 5.**
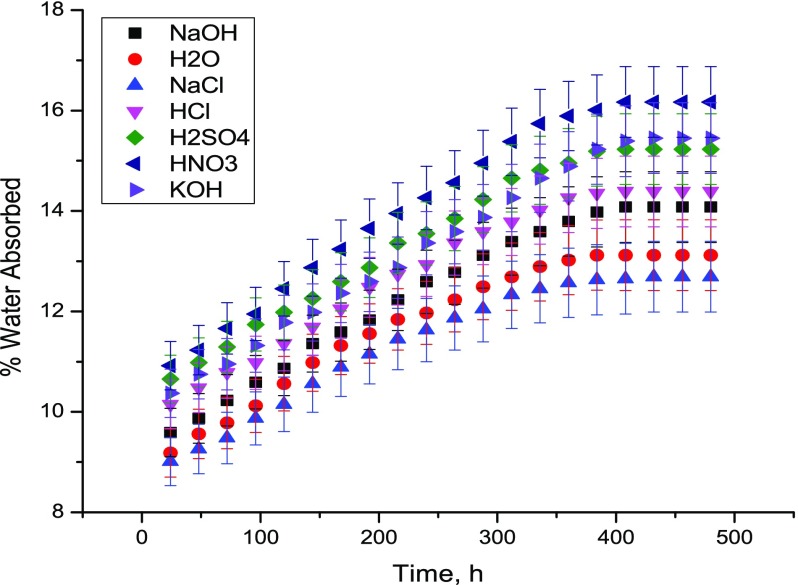
The plots of % weight gain against time for J-EANBr in 10% aq. each of NaCl, HCl, HNO_3_, H_2_SO_4_, NaOH, KOH and distilled water at 30 ± 2 °C.

Water absorption in composites follows Fickian as well as non Fickian mechanism [18]. Assuming unidirectional diffusion in the composite, water absorption in semi-infinite plate exposed on both the sides to the same environment was carried out at room temperature (30 ± 2 °C) according to ASTM-D 570–98 by a change in mass method against distilled water, 10% NaCl, 10% HCl, 10% HNO_3_, 10% H_2_SO_4_, 10% NaOH, and 10% KOH. Water absorbed in the composite with the passage of time was determined according to Equation (4).

(4) $$M=\frac{W_{m}-W_{d}}{W_{d}}\;x\;100$$

where *M* = % water absorbed, *W*_*m*_ = weight of moist sample and *W*_*d*_ = weight of the dry sample. The plot of % water absorbed against time for J-EANBr is presented in Figure 5. Equilibrium time and equilibrium water absorption for J-EANBr are presented in Table 4 from which it is observed that equilibrium time and equilibrium water content for J-EANBr are 384–432 h; 12.7–15.2%, respectively in different environments. The observed equilibrium water content trend is KOH>H_2_SO_4_>HCl>NaOH>H_2_O>NaCl. The nature of the strong electrolytes has affected water structure and hence water absorption tendency. As compared to J-EAN (8.6–12.7%) [16] J-EANBr (12.7–15.2%) showed more water absorption tendency due to its more polar nature.

**Table 4.** Water uptake and diffusivity data of J-EANBr composites at 30 ± 2 °C and in boiling water.

Environment	Equilibrium	% Equilibrium	Diffusivity, 10^−13^
	Time, h	Water content	
H_2_O	384	13.1	2.96
10% HCl	408	14.4	1.72
10% H_2_SO_4_	408	15.2	1.93
10% NaOH	408	14.1	2.06
10% NaCl	432	12.7	2.43
10% KOH	432	15.5	1.14

Note: 11.7% Equilibrium water content in boiling water.

Diffusivity of water in J-EANBr in different environments was determined by determining equilibrium water absorption and initial slopes of *M* against t^1/2^ plots [18]:

(5) $$M=\;\frac{4{\text{M}}_{\text{m}}}{\text{h}}\sqrt{\frac{\text{t}}{\pi }}\;\;\sqrt{{\text{D}}_{\text{X}}}\;$$

(6) $${\text{D}}_{\text{x}}=\;{\left(\frac{\text{h}}{4{\text{M}}_{\text{m}}}\right)}^{2}{\left(\text{slope}\right)}^{2}$$

where *M*_m_ = equilibrium water content, D_x_ = diffusivity, *t* = time (s) and *h* = sample thickness (m). The diffusivity of water in J-EANBr in different environments was determined according to Equations (5) and (6) and reported in Table 4. The observed diffusivity in J-EANBr is H_2_O>NaCl>NaOH>H_2_SO_4_>HCl>KOH. As compared to J – EAN (7.5 × 10^−11^ – 2.9x10^−11^ m^2^s^−1^) J-EANBr (2.96 × 10^−13^–1.14 × 10^−13^ m^2^s^−1^) has shown much lower diffusivity due to its more polar nature. Observed solvation phenomenon in the present case has influenced diffusivity of water in the J-EANBr. Absorption of water in composites causes swelling of fibers till the cell walls are saturated with water and beyond that water exists as free water in the void structure leading to delamination or void formation [19,20]. Absorbed water causes weakening of the interfacial adhesion and hydrolytic degradation of both matrix and fibers and hence deterioration of tensile property [19,20]. Cracking and blistering of fibers cause high water absorption, while degradation causes leaching of small molecules [21,22]. J-EANBr has shown excellent hydrolytic stability even in harsh acidic, alkaline and saline environments confirming its applications in the field of marine.

### 4.5. Water absorption in boiling water

**Figure 6.**
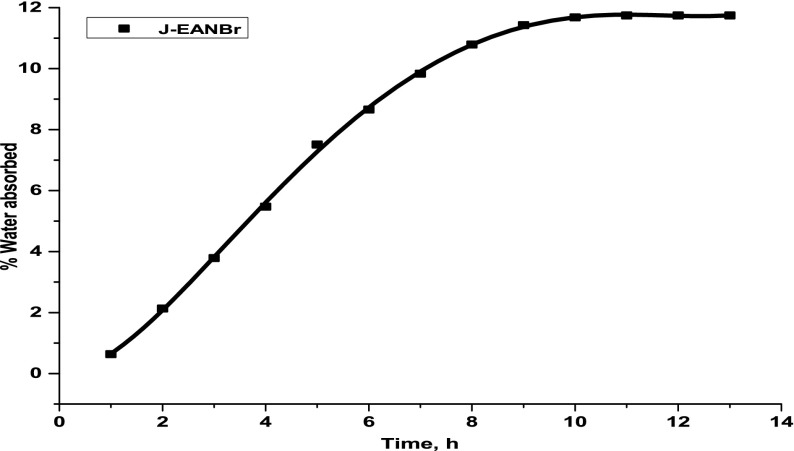
The plots of % weight gain against time for J-EANBr in boiling water.

The water absorption in the J-EANBr was also carried out in boiling water and the % weight gained by the J-EANBr with the passage of time is presented in Figure 6. Observed equilibrium time and equilibrium water absorption for J-EANBr are 11 h; and 11.7%, respectively. Increasing temperature led to drastic reduction in equilibrium time. Water absorption in the composites depends upon various factors such as humidity, temperature, nature of matrix and the reinforcement, etc.

**Scheme 1.**
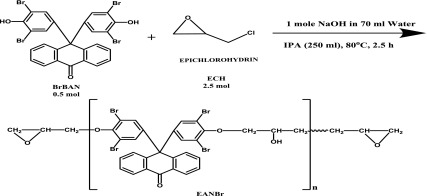
Synthesis of epoxy resin of 9,9′-bis-(3,5-dibromo-4-hydroxyphenyl) anthrone-10 (EANBr).

## 5. Conclusions

Epoxy resin of 9,9′-bis-(3,5-dibromo-4-hydroxyphenyl) anthrone-10 (EANBr) was synthesized and its structure was supported by IR and ^1^HNMR techniques. Thermally and EPK 3251 cured EANBr have shown good thermal stability. J-EANBr has shown good mechanical and electrical properties and excellent hydrolytic stability against harsh environmental conditions. Good thermal, mechanical and electrical properties and excellent hydrolytic stability of J-EANBr has indicated its usefulness as low load bearing housing units, electrical and electronic components and also for marine appliances.

## Funding

This work was supported by University Grants Commission, New Delhi.

## Disclosure statement

No potential conflict of interest was reported by the authors.
